# The Effects of Netarsudil Ophthalmic Solution on Aqueous Humor Dynamics in a Randomized Study in Humans

**DOI:** 10.1089/jop.2017.0138

**Published:** 2018-06-01

**Authors:** Arash Kazemi, Jay W. McLaren, Casey C. Kopczynski, Theresa G. Heah, Gary D. Novack, Arthur J. Sit

**Affiliations:** ^1^ Department of Ophthalmology, Mayo Clinic, Rochester, Minnesota.; ^2^ Aerie Pharmaceuticals, Inc., Bedminster, New Jersey.; ^3^ Aerie Pharmaceuticals, Inc., Durham, North Carolina.; ^4^ Departments of Ophthalmology and Pharmacology, University of California, Davis, California.; ^5^ PharmaLogic Development, Inc., San Rafael, California.

**Keywords:** netarsudil, glaucoma, aqueous humor dynamics, outflow

## Abstract

***Purpose:*** Netarsudil, an inhibitor of Rho kinase and a norepinephrine transporter, has been shown to lower elevated intraocular pressure (IOP) in controlled studies of patients with open-angle glaucoma and ocular hypertension, and in healthy volunteers. The mechanism of this ocular hypotensive effect in humans is unknown.

***Methods:*** The objective of this study was to evaluate the effect of netarsudil 0.02% on aqueous humor dynamics (AHD) parameters. In this double-masked, vehicle-controlled, paired-eye comparison study, 11 healthy volunteers received topical netarsudil ophthalmic solution 0.02% or its vehicle once daily for 7 days (morning dosing). The primary endpoints were the change in AHD parameters, compared between active and vehicle-treated eyes.

***Results:*** In netarsudil-treated eyes, diurnal outflow facility increased from 0.27 ± 0.10 μL/min/mmHg to 0.33 ± 0.11 μL/min/mmHg (+22%; *P* = 0.02) after 7 days of treatment. In placebo-treated eyes, diurnal outflow facility did not significantly change (*P* = 0.94). The difference between netarsudil and placebo eyes in diurnal change of outflow facility was 0.08 μL/min/mmHg (*P* < 0.001). Diurnal episcleral venous pressure (EVP) in netarsudil-treated eyes decreased from 7.9 ± 1.2 mmHg to 7.2 ± 1.8 (-10%; *P* = 0.01). Diurnal EVP was not significantly different between netarsudil- and placebo-treated eyes. There was a trend toward decreasing aqueous humor flow rate (-15%; *P* = 0.08). No treatment changes were seen in uveoscleral outflow rate.

***Conclusions:*** Once-daily dosing of netarsudil ophthalmic solution 0.02% lowered IOP through increasing trabecular outflow facility and reducing EVP. This suggests a combination of mechanisms that affect both the proximal and distal outflow pathways.

## Introduction

A new class of topical agents, Rho kinase (ROCK) inhibitors, has recently been evaluated for reduction of intraocular pressure (IOP) in glaucoma.^1^ Netarsudil, a new chemical entity that inhibits both ROCK and the norepinephrine transporter,^2^ has been shown to lower elevated IOP in controlled studies of patients with open-angle glaucoma and ocular hypertension.^3,4^ In animals, netarsudil lowers IOP by affecting 3 variables of aqueous humor dynamics (AHD): it increases trabecular outflow facility,^5,6^ decreases production of aqueous humor,^6^ and decreases episcleral venous pressure (EVP).^7^ Perfusion of human donor eyes with netarsudil-M1, the active metabolite of netarsudil, has been shown to increase outflow facility and dilate episcleral veins.^8^ However, there have not been any previous studies of the mechanisms of action of netarsudil in living human subjects.

Aqueous humor is produced by the ciliary body, passes through the pupil into the anterior chamber, and exits through both trabecular and uveoscleral pathways.^9^ The aqueous humor flow rate, the resistance to outflow, and the EVP are the main contributors to IOP. Goldmann expressed the relationship between these variables as a simple equation,^10,11^ which was later modified to account for the presence of the uveoscleral outflow pathway (the modified Goldmann equation):

\begin{align*}
IOP = EVP + \frac { 1 }  { C } ( Q - U ) \tag { 1 } 
\end{align*}

where *Q* is the aqueous humor production rate, *C* is outflow facility, and *U* is uveoscleral outflow rate. Measurements of AHD variables have been used to explore the action of pharmacological agents that reduce IOP. For example, measurement of aqueous humor flow rate demonstrated the suppression of aqueous humor production by timolol to lower IOP.^12,13^ Also, calculation of uveoscleral outflow rate from the modified Goldmann equation has been used to demonstrate an increase in uveoscleral outflow rate as a mechanism for IOP reduction in response to prostaglandin analogs.^14,15^

For this study, we evaluated the effects of topically applied netarsudil ophthalmic solution 0.02% on IOP, outflow facility, EVP, aqueous humor flow, and uveoscleral outflow in healthy volunteers.

## Methods

### Study design

This study was designed as a double-masked, vehicle-controlled, paired-eye comparison trial. Study participants included healthy adult volunteers with IOP between 14 and 21 mmHg inclusive in each eye and best-corrected visual acuity (BCVA) in each eye of 20/50 (LogMAR +0.4) or better. Individuals were excluded if they had chronic or acute ophthalmic disease, previous intraocular or refractive surgery, and recent ocular trauma or infection. Individuals with refractive errors greater than −4.00 D or +2.00 D were excluded due to possible effects on measurement of outflow facility using tonography.^16^ Also, women of childbearing potential were excluded if they had a positive urine pregnancy test. The study adhered to the principles of the Declaration of Helsinki and was reviewed and approved by the Institutional Review Board of Mayo Clinic. All subjects gave consent to participate after discussion of risks and benefits of the study.

During a screening examination, heart rate and blood pressure were measured and both eyes were examined by slit-lamp biomicroscopy, including gonioscopy and dilated ophthalmoscopy. IOP was measured by Goldmann applanation tonometry in both eyes. Three-dimensional images of the anterior chamber were recorded by using Scheimpflug photography (Pentacam; Oculus Optikgeräte GmbH, Wetzlar, Germany). BCVA was recorded and individuals were evaluated for their ability to instill eye drops.

Baseline AHD evaluation was scheduled for Day 1 of the study, and enrolled participants instilled 3–8 drops of 2% fluorescein solution (prepared by the Mayo Clinic Research Pharmacy) in each eye approximately 8 h before the start of measurements. Participants arrived at the clinic at 08:00 h, and heart rate, blood pressure, and any ocular symptoms were recorded. Fluorescein concentrations in the anterior chamber and cornea were measured by fluorophotometry^17^ at 09:00, 11:00, and 13:00 h. No other ocular measurements were performed during this time due to the possible effects of topical anesthetics and preservatives on corneal permeability.^18^ After fluorophotometry was completed, IOP, EVP, and outflow facility were measured using the techniques described below. These measurements were repeated at 15:00 h.

The eyes of each participant were then randomized to receive netarsudil ophthalmic solution 0.02% in one eye and its vehicle in the fellow eye (both preserved with 0.015% benzalkonium chloride) each morning for 7 days. Note that while netarsudil was dosed at night in Phase 2b and Phase 3 trials,^4,19^ morning instillation, as used in previous earlier stage trials,^20,21^ was selected for this mechanism study to optimize measurements of AHD during normal working hours. Each participant received 2 bottles of study medication for home use, coded as “right” or “left” eye, but were otherwise externally identical. On the night preceding the Day 8 (posttreatment) examination, participants instilled 2% fluorescein drops as they did in the baseline examination. On the morning of Day 8, participants instilled their 7th dose of study medication in each eye at 06:00 h. Participants returned to the clinic for a repetition of the measurements performed on the baseline day, with the addition of biomicroscopy. Conjunctival hyperemia was scored as 0 (none) to 3 (severe)^19^

### AHD measurements

#### Intraocular pressure

IOP was measured by pneumatonometry (Model 30 Classic; Reichert, Inc., Depew, NY). A mean of 3 measurements was used at each time point.

#### Outflow facility

Outflow facility was measured by using a custom digital Schiøtz tonography unit.^22^ The probe with a 5.5-g weight was held perpendicularly against the anesthetized cornea of the supine subject for 4 min. A second-order polynomial was fitted to pressures recorded by the tonography unit, and outflow facility was directly calculated using Excel spreadsheets (Microsoft, Redmond, WA) based on Grant's formula^23^ and standard tables introduced by Friedenwald.^24,25^

#### Aqueous humor flow rate

Aqueous humor flow rate was calculated by measuring the clearance rate of fluorescein from the anterior chamber and cornea. The concentration of fluorescein was measured by using a scanning ocular fluorophotometer.^26^

#### Episcleral venous pressure

EVP was measured by automated episcleral venomanometry by using a custom-built device and software.^27,28^ A clear silicone inflatable bulb was placed over an episcleral vein, and then inflated at a constant rate. Collapse of the vein was recorded by a video camera. The chamber pressure was recorded and synchronized with the video stream. Brightness profiles across a selected vessel were calculated using custom image analysis software. Peak vessel brightness decreased as increasing pressure in the bulb collapsed the vein. Venous pressure was assumed to be the applied pressure that initiated venous collapse.^28^

#### Uveoscleral outflow rate

Uveoscleral outflow rate was calculated by using equation 1, and measured values for IOP, EVP, outflow facility, and aqueous humor flow rate.

### Statistical analysis

The primary endpoints were the change in AHD parameters, compared between active and vehicle-treated eyes. The sample size was selected to have *a priori* an 80% chance of finding a minimum difference of 1.6 mmHg (α = 0.05, β = 0.20, paired *t*-test), assuming a mean baseline EVP of 6.5 mmHg and standard deviation of 1.5 mmHg, based on previous studies in healthy participants.^27,28^ Observations throughout the day were averaged for “diurnal.”

For AHD parameters, the mean values at baseline were compared with the mean values post-treatment using paired *t*-tests with 2-sided 90% confidence intervals (CIs). In addition, the mean changes in AHD measures were compared between netarsudil and placebo using paired *t*-tests with 2-sided 90% CIs. Probability was estimated by using PC-SAS version 9.3 or higher (Cary, NC). Statistical significance was defined as a one-tailed *P*-value of 0.05 or lower, with no correction for multiplicity.

## Results

### Disposition and demographics

In this paired comparison study conducted between May 2015 and January 2016, from 35 individuals screened, 11 participants were randomized and treated and 10 (90.9%) of them completed the study. Among the individuals screened, but not randomized, 12 did not meet the IOP criteria, 3 were on ocular medications, 2 had suspicious discs, 2 had a history of other eye diseases, 1 was unable to tolerate the AHD measurements, and 4 withdrew consent after screening, but before randomization. One randomized participant discontinued the study early and withdrew consent because of an adverse event (conjunctival hyperemia).

The study population was 90.9% female (10/11), with a mean age of 38.6 ± 13.8 years (range 21–56 years). The majority of participants were white (81.8%, 9/11) and not Hispanic (90.9%, 10/11). Iris color was similarly distributed among brown, blue, and hazel, with one participant with green irides. The eye receiving netarsudil ophthalmic solution 0.02% was the left eye for 6 participants and the right eye for 5 participants.

#### Aqueous humor dynamics

A summary of the AHD findings are presented in Table 1.

**Table 1.** Summary of Mean Diurnal Aqueous Humor Dynamic Measures

		*Netarsudil*		*Vehicle*		*Netarsudil—Vehicle*	
*Measure*	*Day*	*Observed*	*Change from baseline*	*Observed*	*Change from baseline*	*Observed*	*Change from baseline*
IOP (mmHg)	Day 1	17.0 ± 2.5	—	16.7 ± 1.8	—	—	—
	Day 8	12.4 ± 2.2	−4.6 ± 1.8^a^	16.0 ± 2.1	−0.7 ± 0.8^b^	−3.5^a^	−3.9^a^
Outflow facility (μL/min/mmHg)	Day 1	0.27 ± 0.10	—	0.30 ± 0.12		—	—
	Day 8	0.33 ± 0.11	0.05 ± 0.07^c^	0.27 ± 0.12	−0.03 ± 0.06	0.06^b^	0.08^a^
EVP (mmHg)	Day 1	7.90 ± 1.24	—	7.83 ± 1.70	—	—	—
	Day 8	7.21 ± 1.75	−0.69 ± 0.84^c^	7.49 ± 1.99	−0.35 ± 1.00	−0.28	−0.35^d^
Aqueous humor flow rate (μL/min)	Day 1	2.53 ± 0.90	—	2.40 ± 0.48	—	—	—
	Day 8	2.13 ± 0.41	−0.39 ± 0.08^d^	2.24 ± 0.49	−0.16 ± 0.20	−0.11	−0.24^d^
Uveoscleral outflow rate (μL/min)	Day 1	0.08 ± 1.19	—	−0.18 ± 1.42	—	—	—
	Day 8	0.44 ± 0.92	0.36 ± 1.10	−0.02 ± 1.14	0.16 ± 0.82	0.46^d^	0.20

Presented as mean ± standard deviation.

*N* = 10 for all observations with the exception of outflow facility at 15:00 h.

^a^ *P* < 0.001.

^b^ *P* < 0.01.

^c^ *P* < 0.05.

^d^ *P* < 0.1.

“Netarsudil–Vehicle” refers to a comparison between eyes receiving netarsudil and those receiving vehicle.

Mean diurnal values are presented for IOP (Days 1 and 8), outflow facility (Days 1 and 8), EVP (Days 1 and 8), and uveoscleral outflow (Days 1 and 8).

EVP, episcleral venous pressure; IOP, intraocular pressure.

#### Intraocular pressure

Observed values: At baseline, mean IOP in the eyes randomized to netarsudil and placebo were similar: 17.4 ± 2.9 and 17.2 ± 2.2 mmHg, respectively, at 13:00 h and 16.6 ± 2.4 and 16.1 ± 1.6 mmHg, respectively, at 15:00 h. Baseline mean diurnal IOP (average of IOP at 13:00 and 15:00) was 17.0 ± 2.5 and 16.7 ± 1.8 mmHg, respectively. After 7 days of dosing with netarsudil, with the last dose at 06:00 h on Day 8, mean IOP decreased to 12.8 ± 2.7 mmHg and 12.0 ± 1.9 mmHg at 13:00 h (7 h after dosing) and 15:00 h (9 h after dosing), respectively. Mean diurnal IOP in netarsudil-treated eyes decreased to 12.4 ± 2.2 mmHg. In placebo-treated eyes, mean IOP decreased to 16.4 ± 2.6 mmHg and 15.5 ± 1.8 mmHg, respectively. Mean diurnal IOP in placebo-treated eyes decreased to 16.0 ± 2.1 mmHg.

Treatment effects: On Day 8 (the 7th day of treatment), mean IOP in netarsudil-treated eyes was 3.6 mmHg and 3.5 mmHg lower than in placebo-treated eyes at 13:00 and 15:00 h, respectively (*P* < 0.0001), and the mean diurnal IOP was 3.5 mmHg lower (*P* < 0.0001) than in placebo-treated eyes. Mean IOP on Day 8 was 4.6 mmHg lower than baseline at 13:00 and 15:00 h (*P* < 0.0001) in netarsudil-treated eyes. Reductions in mean IOP of less than 1 mmHg were recorded in placebo-treated eyes at 13:00 and 15:00 h. However, the mean IOP decrease from baseline in the netarsudil-treated eyes was greater than in the placebo-treated eyes by 3.8 mmHg and 3.9 mmHg at 13:00 and 15:00 h, respectively (*P* < 0.0001).

#### Aqueous outflow facility by tonography

Observed values: At baseline, mean aqueous outflow facility in the eyes randomized to netarsudil and placebo were similar: 0.29 ± 0.13 and 0.30 ± 0.11 μL/min/mmHg at 13:00 h and 0.28 ± 0.092 and 0.31 ± 0.14 μL/min/mmHg at 15:00 h, respectively. Baseline mean diurnal outflow facility was 0.27 ± 0.10 and 0.30 ± 0.12 μL/min/mmHg for netarsudil- and placebo-treated eyes, respectively. After 7 days of dosing with netarsudil, mean outflow facility at 13:00 and 15:00 h increased to 0.32 ± 0.11 μL/min/mmHg and 0.33 ± 0.11 μL/min/mmHg, respectively, and mean diurnal outflow facility increased to 0.33 ± 0.11 μL/min/mmHg. In placebo-treated eyes, mean outflow facility decreased to 0.28 ± 0.13 μL/min/mmHg and 0.26 ± 0.12 μL/min/mmHg at 13:00 and 15:00 h, respectively, and mean diurnal outflow facility in placebo-treated eyes decreased to 0.27 ± 0.12 μL/min/mmHg.

Treatment effects: On Day 8, the mean outflow facility in netarsudil-treated eyes was 0.04 μL/min/mmHg (*P* = 0.03) and 0.08 μL/min/mmHg (*P* = 0.001) higher than in placebo-treated eyes at 13:00 and 15:00 h, respectively, and the mean diurnal outflow facility was 0.06 μL/min/mmHg higher (*P* = 0.004) than in placebo-treated eyes. The mean increase in outflow facility on Day 8 compared with baseline for netarsudil-treated eyes was 0.03 ± 0.11 μL/min/mmHg (*P* = 0.2) and 0.07 ± 0.10 μL/min/mmHg (*P* = 0.049) at 13:00 and 15:00 h, respectively, and mean diurnal change from baseline outflow facility was 0.05 ± 0.07 μL/min/mmHg (*P* = 0.02). For placebo-treated eyes, there was no statistically significant change in outflow facility from baseline at 13:00 and 15:00 h (-0.02 ± 0.09 μL/min/mmHg and −0.04 ± 0.06 μL/min/mmHg, respectively; *P* = 0.8 and *P* = 0.9), or diurnally (-0.03 ± 0.06 μL/min/mmHg; *P* = 0.9). The change in outflow facility from baseline in the netarsudil-treated eyes was significantly greater than the change in the placebo-treated eyes at 13:00 and 15:00 h (0.05 μL/min/mmHg and 0.1 μL/min/mmHg, respectively; *P* = 0.03 and *P* = 0.01), and diurnally (0.08 μL/min/mmHg; *P* < 0.001).

#### EVP by venomanometry

Observed values: At baseline, mean EVP in the eyes randomized to netarsudil and placebo were similar: 8.0 ± 1.3 mmHg and 7.9 ± 1.8 mmHg at 13:00 h and 7.8 ± 1.4 mmHg and 7.8 ± 1.7 mmHg at 15:00 h, respectively. Baseline mean diurnal EVP in the eyes randomized to netarsudil and placebo was 7.9 ± 1.2 mmHg and 7.8 ± 1.7 mmHg, respectively. After 7 days of dosing with netarsudil, mean EVP at 13:00 and 15:00 h decreased to 7.3 ± 1.8 mmHg and 7.1 ± 1.7 mmHg, respectively. Mean diurnal EVP in netarsudil-treated eyes decreased to 7.2 ± 1.7 mmHg. In placebo-treated eyes, mean EVP decreased to 7.6 ± 2.0 mmHg and 7.3 ± 2.0 mmHg, respectively. Mean diurnal EVP in placebo-treated eyes decreased to 7.5 ± 2.0 mmHg.

Treatment effects: After 7 days of treatment (Day 8), there was no statistically significant difference between netarsudil- and placebo-treated eyes in mean EVP at 13:00 and 15:00 h (−0.4 and −0.2 mmHg, respectively; *P* = 0.3 and 0.4), or mean diurnal EVP (−0.3 mmHg; *P* = 0.4). However, EVP in netarsudil-treated eyes on Day 8 was decreased significantly compared to baseline at 13:00 h (−0.6 ± 1.0 mmHg; *P* = 0.046) and diurnally (−0.7 mmHg; *P* = 0.01), with a trend toward a decrease at 15:00 h (-0.8 ± 1.0 mmHg; *P* = 0.06). There was no statistically significant change in EVP in placebo-treated eyes compared to baseline at 13:00 h, 15:00 h, or diurnally (−0.3 ± 1.3 mmHg, −0.4 ± 0.9 mmHg, and −0.3 mmHg; *P* = 0.3, *P* = 0.09, and *P* = 0.2, respectively). The difference in change from baseline EVP between netarsudil- and placebo-treated eyes was −0.5 mmHg (*P* = 0.06) and −0.2 mmHg (*P* = 0.3) at 13:00 and 15:00 h, respectively, and the difference in mean diurnal change from baseline EVP was −0.3 mmHg (*P* = 0.08).

#### Aqueous humor flow rate by fluorophotometry

Observed values: At baseline, mean aqueous humor flow rate in the eyes randomized to netarsudil and placebo was similar: 2.5 ± 0.9 and 2.4 ± 0.5 μL/min, respectively. On Day 8, mean aqueous humor flow rate was 2.1 ± 0.4 μL/min in netarsudil-treated eyes and 2.2 ± 0.5 μL/min in placebo-treated eyes.

Treatment effects: There was no significant difference in aqueous humor flow rate between netarsudil- and placebo-treated eyes on Day 8, after 7 days of treatment (0.1 μL/min; *P* = 0.3). On Day 8, there was a trend toward a decrease in aqueous humor flow rate relative to baseline in netarsudil-treated eyes (−0.4 ± 0.8 μL/min; *P* = 0.08), with no trend in placebo-treated eyes (−0.2 ± 0.6 μL/min; *P* = 0.2). There was a trend toward a difference in change from baseline aqueous humor flow rate between netarsudil- and placebo-treated eyes (−0.2 μL/min; *P* = 0.08).

#### Uveoscleral outflow rate calculated by modified Goldmann's equation

Observed values: At baseline, calculated mean uveoscleral outflow rate was higher in the eyes randomized to netarsudil compared to placebo, −0.1 ± 1.2 and −0.4 ± 1.4 μL/min at 13:00 h and 0.2 ± 1.4 and −0.1 ± 1.6 μL/min at 15:00 h, respectively. Calculated baseline mean diurnal uveoscleral outflow rate was 0.1 ± 1.2 for netarsudil and −0.2 ± 1.4 μL/min for placebo. It is worth noting that negative values for uveoscleral outflow are not physiological. After 7 days of dosing with netarsudil, calculated mean uveoscleral outflow at 13:00 and 15:00 h was 0.3 ± 1.0 μL/min and 0.5 ± 1.0 μL/min, respectively, and calculated mean diurnal uveoscleral outflow rate was 0.4 ± 0.9 μL/min. In placebo-treated eyes on Day 8, calculated mean uveoscleral outflow rate was to −0.2 ± 1.2 μL/min and 0.2 ± 1.2 μL/min at 13:00 and 15:00 h, respectively, and calculated mean diurnal uveoscleral outflow rate was 0.0 ± 1.1 μL/min.

Treatment effects: The difference between netarsudil- and placebo-treated eyes in calculated mean uveoscleral outflow rate was 0.5 μL/min (*P* = 0.04) and 0.4 μL/min (*P* = 0.1) at 13:00 and 15:00 h, respectively, and the difference in mean diurnal uveoscleral outflow was 0.5 μL/min (*P* = 0.07). Although there was a statistically significant difference between netarsudil- and placebo-treated eyes at 13:00 h on Day 8, it should be noted that calculated mean uveoscleral outflow was higher in the eyes assigned to netarsudil treatment at baseline. There was no significant difference in calculated uveoscleral outflow for netarsudil or placebo on Day 8 relative to respective baseline values. Changes from baseline in calculated mean uveoscleral outflow for netarsudil were 0.5 ± 1.0 μL/min (*P* = 0.08) and 0.4 ± 1.5 μL/min (*P* = 0.2) at 13:00 and 15:00 h, respectively, and mean diurnal change from calculated baseline uveoscleral outflow was 0.4 ± 1.1 μL/min (*P* = 0.2). There was no significant change from baseline in calculated mean uveoscleral outflow for placebo at 13:00 h (0.2 ± 0.8 μL/min; *P* = 0.3), 15:00 h (0.1 ± 1.1 μL/min; *P* = 0.4), or diurnally (0.2 ± 0.8 μL/min; *P* = 0.3). Similarly, there was no significant difference in calculated change from baseline uveoscleral outflow between netarsudil- and placebo-treated eyes at 13:00 h (0.3 μL/min; *P* = 0.09), 15:00 h (0.3 μL/min; *P* = 0.2), or diurnally (0.2 μL/min; *P* = 0.2).

#### Safety

Each participant reported mild or, in one case moderate, conjunctival hyperemia in the netarsudil-treated eye, and mild conjunctival hyperemia was observed on slit-lamp examination. One participant reported mild dry eye in the netarsudil-treated eye (assessed as related to netarsudil use) and one participant reported mild eye pruritus in the netarsudil-treated eye (assessed as likely unrelated to netarsudil use). No adverse events were reported in the vehicle-treated eye. There were no changes in visual acuity, heart rate, or blood pressure.

## Discussion

The objective of this study was to evaluate the effects of netarsudil ophthalmic solution 0.02% on AHD. While this question has been studied in rabbits, mice, non-human primates, and human donor eyes,^5,6,29,30^ this study represents the first measurement of netarsudil effects on AHD in living humans. The methods required to measure these variables in human subjects are technically challenging, and are necessarily noninvasive. In contrast, some of the methods used to measure AHD in animals are invasive (eg, cannulation of the episcleral veins and histological quantification of tracers) and cannot be used in humans.^7,31^

In this study, netarsudil decreased IOP by 4.6 mmHg from baseline (∼27%). In eyes receiving the vehicle, there was a small reduction in IOP (0.7 mmHg). Given that this was a paired-eye study, a small contralateral effect of the drug cannot be ruled out, although this seems unlikely given that systemic exposure to netarsudil is not measurable after topical ocular dosing.^21^ The ocular hypotensive effect of netarsudil measured in this study was similar to that observed in a previous study in healthy volunteers following 8 days of once-daily dosing,^21^ and therefore confirms the ability of netarsudil to produce large reductions in IOP in participants with normal baseline IOP.

The aqueous humor dynamic variables measured in this study suggest that an increase in outflow facility was the most important parameter to reduce IOP in response to netarsudil. In netarsudil-treated eyes, outflow facility increased by approximately 20% both from baseline and compared to the contralateral placebo-treated eyes. This result is consistent with the ability of the netarsudil primary metabolite, AR-13503, to increase outflow facility in perfused enucleated human eyes,^5^ and the ability of netarsudil,^6^ as well a number of other Rho kinase inhibitors,^32,33,29^ to increase outflow facility in non-human primates. However, based upon the Goldmann equation, a 20% increase in outflow facility accounts for less than half of the measured decrease in IOP produced by netarsudil, if all other parameters remained unchanged. Other changes in dynamic variables must account for the remaining change in IOP.

Measurement of EVP by using an objective noninvasive technique showed that netarsudil reduced EVP by about 10% from baseline. However, EVP was not different between netarsudil- and placebo-treated eyes. This may have been due to higher variability in measurement of EVP in this study than anticipated (up to SD = 1.81 in some groups vs. 1.5 mmHg expected), resulting in the study having lower power than estimated. In a previous study by Kiel and Kopczynski, topical netarsudil was shown to reduce EVP in normotensive rabbits by approximately 35% from baseline.^7^ The larger effect seen in rabbits could reflect a species difference, and it was noted in that study of Dutch belted rabbits that the baseline IOP (33.2 ± 8.3 mmHg) and EVP (16.3 ± 3.4 mmHg) were much higher than in New Zealand White rabbits (16.2 ± 4.5 mmHg and 10.4 ± 2.5 mmHg, respectively). It is also possible that the difference in effect could be attributed to differences in the method of measurement. Cannulation of the episcleral veins in rabbits enables a more direct measurement of pressure, as well as a continuous tracing of the pressure profile. However, this technique would require removal of the overlying conjunctiva and penetration of the vessel with a glass cannula. These procedures could potentially affect absorption of netarsudil into the episcleral vasculature. Concerning the mechanism for EVP reduction, it is not clear if the effect on EVP is simply caused by vessel dilation, since dilation of the arteriovenous anastomoses in the episcleral vasculature could increase blood flow to the episcleral veins and offset the pressure reduction expected from episcleral vein dilation.^34^ Regardless, these results suggest an effect of netarsudil on the distal portion of the conventional outflow pathway.

Measurements of aqueous humor flow rate by fluorophotometry showed a trend toward a decrease in flow rate in netarsudil-treated eyes compared to baseline. While this trend was not statistically significant (*P* = 0.08), this study was not powered to detect a change in flow rate of this magnitude. However, the numerical decrease in aqueous humor flow rate of about 15% relative to baseline was consistent with a previous study in normotensive monkeys, which reported 23% decrease in flow rate from baseline in eyes treated with topical netarsudil.^6^ As with the measurement of EVP, the larger effect seen in the animal model could reflect a species difference or a difference in methodology. Of note, that study used a higher concentration of netarsudil (0.04% vs. 0.02% in our study), and 2 drops (instead of a single drop) were given in each eye. Nevertheless, further research is required to clarify the effect of netarsudil on aqueous humor flow rate in humans.

Uveoscleral outflow rate was not directly measured in this study, but was instead calculated from the modified Goldmann equation and the measured values for IOP, outflow facility, EVP, and aqueous humor flow rate. Unfortunately, direct measurement of uveoscleral outflow rate in humans requires perfusion with radioactive tracers and subsequent enucleation, a technique that has only been performed in a small group of patients with diseased eyes scheduled for enucleation.^35^ Since uveoscleral outflow rate in our study was calculated, rather than measured directly, its accuracy and variability are dependent on the accuracy and variability of the parameters of AHD that can be directly measured. In a small study such as ours, this can lead to calculation of negative values, which we reported for some subjects at baseline and are obviously nonphysiological. However, uveoscleral outflow rates for normal human eyes are poorly understood and values reported have varied widely.^36^ As a percent of aqueous humor production, uveoscleral flow rates have ranged from less than 10%^37,38^ to as high as 85% at night.^37^ There was a trend toward an increase in uveoscleral outflow rate in netarsudil-treated eyes at one measurement time (13:00 h), but none of the other changes approached statistical significance. Given the variability in this parameter, and the small number of subjects in this study, any apparent change in uveoscleral outflow rate should be interpreted with caution.

Overall, there appear to be multiple mechanisms by which netarsudil lowers IOP. No single parameter is able to explain the changes in IOP even when extrapolating to the limits of the 95% confidence intervals for the AHD parameters. This contrasts with other agents, such as topical β-adrenoceptor antagonists, which are known to lower IOP through a single mechanism, an approximately 50% reduction of aqueous humor production.^12,13^ The 2 parameters that demonstrated statistically significant changes from baseline, outflow facility, and EVP could explain the observed changes in IOP. However, trends suggesting a reduction in aqueous humor flow rate and an increase in uveoscleral outflow rate require further research.

There were no substantial safety issues with netarsudil in this study, other than the previously reported finding of conjunctival hyperemia, which was mostly mild and transient.^3,4,21^

In conclusion, in this study of healthy volunteers, once-daily dosing of netarsudil ophthalmic solution 0.02% lowered IOP through multiple mechanisms of action, including increased trabecular outflow facility and decreased EVP. This appears to be a unique combination of mechanisms that impact both the proximal and distal portions of the conventional outflow pathway. The roles of aqueous humor suppression and uveoscleral outflow enhancement require further investigation.
